# The skin transcriptome in hidradenitis suppurativa uncovers an antimicrobial and sweat gland gene signature which has distinct overlap with wounded skin

**DOI:** 10.1371/journal.pone.0216249

**Published:** 2019-05-06

**Authors:** Margaret Coates, Paula Mariottoni, David L. Corcoran, Hélène Fradin Kirshner, Tarannum Jaleel, David A. Brown, Stephen R. Brooks, John Murray, Maria I. Morasso, Amanda S. MacLeod

**Affiliations:** 1 Department of Dermatology, Duke University, Durham, NC, United States of America; 2 Duke Center for Genomic and Computational Biology, Duke University, Durham, NC, United States of America; 3 Department of Surgery, Duke University, Durham, NC, United States of America; 4 National Institute of Arthritis and Musculoskeletal and Skin Diseases, National Institute of Health, Bethesda, MD, United States of America; 5 Department of Immunology, Duke University, Durham, NC, United States of America; 6 Pinnell Center for Investigative Dermatology, Duke University, Durham, NC, United States of America; NYU Langone Medical Center, UNITED STATES

## Abstract

Hidradenitis suppurativa (HS) is a debilitating chronic inflammatory skin disease resulting in non-healing wounds affecting body areas of high hair follicle and sweat gland density. The pathogenesis of HS is not well understood but appears to involve dysbiosis-driven aberrant activation of the innate immune system leading to excessive inflammation. Marked dysregulation of antimicrobial peptides and proteins (AMPs) in HS is observed, which may contribute to this sustained inflammation. Here, we analyzed HS skin transcriptomes from previously published studies and integrated these findings through a comparative analysis with a published wound healing data set and with immunofluorescence and qPCR analysis from new HS patient samples. Among the top differently expressed genes between lesional and non-lesional HS skin were members of the *S100* family as well as *dermcidin*, the latter known as a sweat gland-associated AMP and one of the most downregulated genes in HS lesions. Interestingly, many genes associated with sweat gland function, such as *secretoglobins* and *aquaporin 5*, were decreased in HS lesional skin and we discovered that these genes demonstrated opposite expression profiles in healing skin. Conversely, HS lesional and wounded skin shared a common gene signature including genes encoding for S100 proteins, defensins, and genes encoding antiviral proteins. Overall, our results suggest that the pathogenesis of HS may be driven by changes in AMP expression and altered sweat gland function, and may share a similar pathology with chronic wounds.

## Introduction

### Hidradenitis suppurativa is a multifactorial disease characterized by chronic inflammatory non-healing sinus tracts leading to impaired quality of life

Hidradenitis suppurativa (HS) is characterized by recurrent painful nodules, abscesses and sinus tract formation leading to chronic non-healing wounds [1, 2]. Cutaneous nodules form, which over time may rupture, resulting in painful, deep dermal abscesses. With disease progression, draining sinus tracts, open wounds, fibrosis, and scarring can be observed, which together can often accompany significant disfigurement, in addition to pain, malodor, and drainage. HS is common; it is estimated that 1–4% of the general population is affected and this percentage varies by geographic region with disproportionately more females, young adults, and African Americans and biracial patients being affected [3, 4]. Overall, chronic wounds are a source of acute and chronic infections, chronic pain and have a profound negative impact on activities of daily living. This negative impact on quality of life has been found to have an impact similar to that seen in patients with renal and heart failure [5, 6].

Improved knowledge of immune response dysregulation in HS, including innate antimicrobial immunity, may unveil mechanisms of disease pathogenesis and may ultimately help develop therapies that lead to better disease outcome for patients. This understanding could lead to better healing, decreased risk of infection, and ultimately improved quality of life for those with HS. In this manuscript, we use a transcriptomics-centered approach to investigate the pathogenesis of HS, uncovering a wide range of gene expression changes that may cause disease.

The understanding of the inflammatory and antimicrobial processes in HS has begun but is incomplete [7–16]. Like chronic non-healing wounds such as chronic venous ulcers and diabetic ulcers, HS lesions are often associated with dysregulated immune responses including altered expression of various cytokines, chemokines and antimicrobial proteins (AMPs) [9, 17]. As effectors of innate immunity, AMPs act as endogenous microbicidals against invading microbes [18–23]. AMPs directly kill Gram-positive and Gram-negative bacteria, fungi, and certain viruses [24, 25]. Over- or under-expression of AMPs has been implicated in various cutaneous diseases, such as psoriasis, atopic dermatitis, and in chronic leg ulcers [26–28]. Although efforts have been made to begin characterizing AMP expression in HS, our understanding is very limited.

It is important to note that HS is thought to be primarily due to occlusion and subsequent inflammation of the hair follicle and not the sweat gland itself [1]. However, in about 50% of all HS patients, inflammation and/or secondary involvement of the sweat gland unit is observed, especially after rupture of dilated hair follicles and half of patients with HS report a change in their sweating behavior before an overt lesion occurs [2, 29, 30]. This raises the possibility that changes in sweat gland-associated AMPs may be functionally linked to HS. Consequently, a better understanding of the molecular and cellular mechanisms involved in HS antimicrobial immunity and the impact of the sweat gland and its antimicrobial products on inflammation and wound healing is needed to ultimately develop optimized therapies for HS.

Dysregulation of host innate antimicrobial immunity and sweat gland pathology could play a significant role in the inflammatory response in HS. However, this relationship has not yet been sufficiently characterized. In this manuscript, we employ first a biocomputational approach to examine the relationship between HS and innate antimicrobial defenses using a previously published data set (GSE72702) [14]. Additionally, we analyzed the gene expression signatures of a skin wound RNA-seq data set (GSE97615) [31]. Using differently expressed genes (DEGs) from both data sets, we show that HS lesional skin and skin wounds share distinct DEGs, demonstrating that HS and wounded skin have a common gene signature underlying possibly a common pathogenesis pathway. We further identified that expression of multiple AMPs and key sweat gland-associated genes were downregulated in HS lesional skin compared to non-lesional skin. Conversely, expression of many inflammation-associated AMPs was increased in HS lesional skin, confirming previous studies examining the roles of AMPs in HS and further adding to our understanding of this disease [32]. Increased expression of multiple S100 family members was confirmed by qPCR and immunofluorescence staining of donor-matched HS lesional and non-lesional samples. We also confirmed by qPCR and IF the decrease in expression of dermcidin (DCD), a key sweat gland-associated AMP and one of the most downregulated genes in HS lesions identified by the biocomputational approach. Further, analysis of microarray GSE72702 and IF on new HS samples verified that expression of multiple other genes associated with sweat gland function are decreased in HS lesional skin. Finally, we describe a common gene expression signature in HS lesions and wounded skin. A number of AMPs have increased expression in both HS lesions and wounded skin. Conversely, *DCD*, as well as many sweat gland-associated genes were greatly suppressed in lesional HS skin but highly upregulated in healing skin.

Overall, our results suggest that the pathogenesis of HS may be driven by changes in AMP expression, altered sweat gland function, and may also share a similar pathology with wounds.

## Materials and methods

### Analysis of gene expression data sets

#### Microarray data set

We used the publicly available microarray dataset from Blok *et al*. (GEO accession number: GSE72702) to evaluate changes in gene expression between lesional versus non-lesional skin in HS patients [14]. This dataset results from mRNA microarray experiments performed on skin biopsy samples from patients with HS. The samples were split between lesional skin (n = 17) and healthy non-lesional skin obtained from the upper arm or leg (n = 13). RNA was hybridized to the GeneChip HT HG-U133+PM Array (Affymetrix, Santa Clara, CA, U.S.A.). We used the normalized dataset submitted to the Gene Expression Omnibus [33]. The data had been previously normalized by Robust Multi-array Average (RMA) using ArrayStudio software version 8.0 (OmicSoft Corp., St Morrisville, NC, U.S.A.). To identify differentially expressed genes (DEGs) between the lesional and non-lesional samples we first removed the 4 lesional skin samples for which no matched healthy non-lesional skin sample had been obtained. Also, prior to analysis we filtered lowly-expressed and invariant microarray probe sets, i.e. those with an expression level < 4 in all but two samples or a standard deviation < 0.1 across all samples. After filtering, the dataset consisted of 26 (13 paired lesional and non-lesional) samples and 51,567 probe sets.

To identify DEGs between lesional and non-lesional samples, we used the R package *nlme* to implement a mixed-effects model including the patient ID as the random effect [34]. P-values were corrected for multiple testing using the Benjamini-Hochberg method [35]. Significantly changing genes were defined as probe sets with an adjusted p-value < 0.05.

To identify genes whose expression varies in similar fashion to the 1553946_PM_at probe (corresponding to the *DCD* gene), we calculated the Pearson correlation between the 1553946_PM_at probe and all other probes in the dataset across all samples using the R statistical programming environment.

#### RNA-seq data set

We used the publicly available RNA-seq dataset from Iglesias-Bartolome *et al*. (GEO accession number: GSE97615) to identify DEGs between wounded and non-wounded human skin samples [31]. This dataset contained human axillary skin wounds at baseline (Day 1, unwounded), two days after full-thickness 3-mm punch biopsy wounding (Day 3), and five days after wounding (Day 6). The raw data was reprocessed as initially described in Iglesias-Bartolome *et al*. using the Partek Genomics Suite Analysis Toolkit version 6.6 (www.partek.com) to generate read counts per gene. In brief, reads were aligned to the hg19 version of the human genome using the TopHat v2.1 alignment tool, and expression was quantified by the Partek E/M algorithm based on known RefSeq transcripts [31]. Genes that did not have at least 10 reads in any one skin sample were removed from subsequent analysis, resulting in a data set of 12 samples and 26,473 genes. To identify genes that change across time we calculated the moderated F-statistic in limma using voom to estimate the mean-variance relationship [36, 37]. P-values were corrected for multiple testing using the Benjamini-Hochberg method. Genes were considered differentially expressed if the adjusted p-value was < 0.05. The DEGs were then compared against those identified as differentially expressed (adjusted p-value < 0.05) in our analysis of the Blok *et al*. lesion/non-lesion microarray data of HS samples.

### Heatmaps

Heatmaps were generated using the R package *pheatmap* [38]. For data visualization, probe sets were z-score transformed and capped when the absolute scaled values exceeded 2.5. Genes and samples were clustered using a correlation distance with complete linkage.

### Preparation of skin samples

All qPCR analyses and immunofluorescence on HS samples as reported in this manuscript were performed using samples from skin punch biopsies (4-mm) of clinically affected, “lesional” skin obtained from patients visiting a dermatologist at Duke University Medical Center Dermatology Clinic. Clinically unaffected, but adjacent, “non-lesional” biopsies were also obtained. Written informed consent was obtained from all patients for participation in the study. This tissue was obtained in accordance with the Duke Health Institutional Review Board (IRB) protocol 0007979, "Immune Signaling in Psoriasis and other Immune-mediated Diseases". De-identified normal skin samples were obtained from surgical skin waste, in accordance with the Duke Health IRB protocol 00090566, "Access to de-identified skin samples". Biopsies for immunohistochemistry were immediately placed in Tissue-Tek O.C.T Compound (Sakura Finetek USA) and stored at -80°C. For future RT-qPCR, samples were homogenized by mincing into small pieces with surgical scissors, lysed in TRIzol Reagent (ThermoFisher, Waltham, MA) and stored at -80°C for RNA isolation.

### Real-time polymerase chain reaction (qPCR)

RNA extraction was performed using the Direct-zol RNA Purification Kit (Zymo Research, Tustin, CA). cDNA was synthesized using iScript cDNA Synthesis Kit (Bio-Rad, Hercules, CA). qPCR was performed for determining gene expression using Fast SYBR Green Master Mix (ThermoFisher, Waltham, MA) and primers specific for DCD, S100A7, S100A8, and S100A7A (Integrated DNA Technologies, Skokie, IL) (see **Table 1**) on a StepOnePlus Real-Time PCR machine (Applied Biosystems, Foster City, CA). PCR was performed for 40 cycles with a melting temperature of 95°C for 3 seconds and an annealing/extension temperature of 60°C for 30 seconds. qPCR was performed on 6 (3 paired lesional and non-lesional) samples. All data was normalized to the average gene expression levels of HS non-lesional skin using the comparative ΔΔ C_T_ method [39].

**Table 1:** Primer sequences and melting temperatures.

Primer	Sequence	Tm (°C)
hGAPDH fwd	CAAGAGCACAAGAGGAAGAGAG	55
hGAPDH rev	CTACATGGCAACTGTGAGGAG	55.3
hDCD fwd	AAAGCCAAGGAAGCAGAGAT	54.3
hDCD rev	CTCCTTTACCCACGCTTTCT	54.7
hS100A7 fwd	CCTGCTGACGATGATGAA	52
hS100A7 rev	TGGCTCTGCTTGTGGTAG	54.6
hS100A8 fwd	AGTGTCCTCAGTATATCAG	47.5
hS100A8 rev	CTCTTTGTGGCTTTCTTC	48.3
hS100A7A fwd	GCTGACGATGATGAAGGAGAAC	55.5
hS100A7A rev	CAGTGGCGAGGTAATGTATGC	55.9

#### Statistical analysis

To determine fold change (FC) in gene expression for genes examined via qPCR, statistical analysis was performed using the Student’s t-test with p-value < 0.05. Data are shown as mean +/- standard error of the mean. Analysis was performed in GraphPad Prism v8.0 (GraphPad Software, La Jolla, CA).

### Immunofluorescence

Samples in Tissue-Tek O.C.T Compound (Sakura Finetek, Torrance, CA) were sectioned and fixed in 4% paraformaldehyde. Staining was performed using an established immunohistochemistry protocol [40]. Samples were permeabilized in 0.1% Triton X (Millipore-Sigma, St. Louis, MO) for 10 minutes. Samples were incubated with monoclonal mouse anti-human S100A7 antibody (Clone 47C1068, 1:200 dilution, ThermoFisher, Waltham, MA), monoclonal mouse anti-human S100A8 antibody (Clone CF-145, 1:500 dilution, ThermoFisher, Waltham, MA), monoclonal mouse anti-human DCD antibody (Clone G-81, 1:50 dilution, Santa Cruz Biotechnology, Dallas, TX), monoclonal mouse anti-human K77 antibody (Clone AE1/AE3, 1:250 dilution, Abcam, San Francisco, CA), or unlabeled IgG control (Southern Biotech, Birmingham, AL) overnight at 4°C. Samples were then washed with 0.01% Triton-X and incubated with Cy3-congugated anti-IgG secondary antibody (Invitrogen, Waltham, MA) for 45 minutes. Secondary antibody was used as control for all DCD and K77 co-stained samples. Nuclear counterstaining was performed with DAPI. Images were acquired using the IX73 inverted microscope (Olympus, Center Valley, PA). Staining of all directly-compared specimens was performed using the same antibody concentrations and exposure times were kept consistent throughout samples.

## Results

### Genes related to humoral immunity, AMPs, and response to bacterium are upregulated in HS lesions

**Fig 1:**
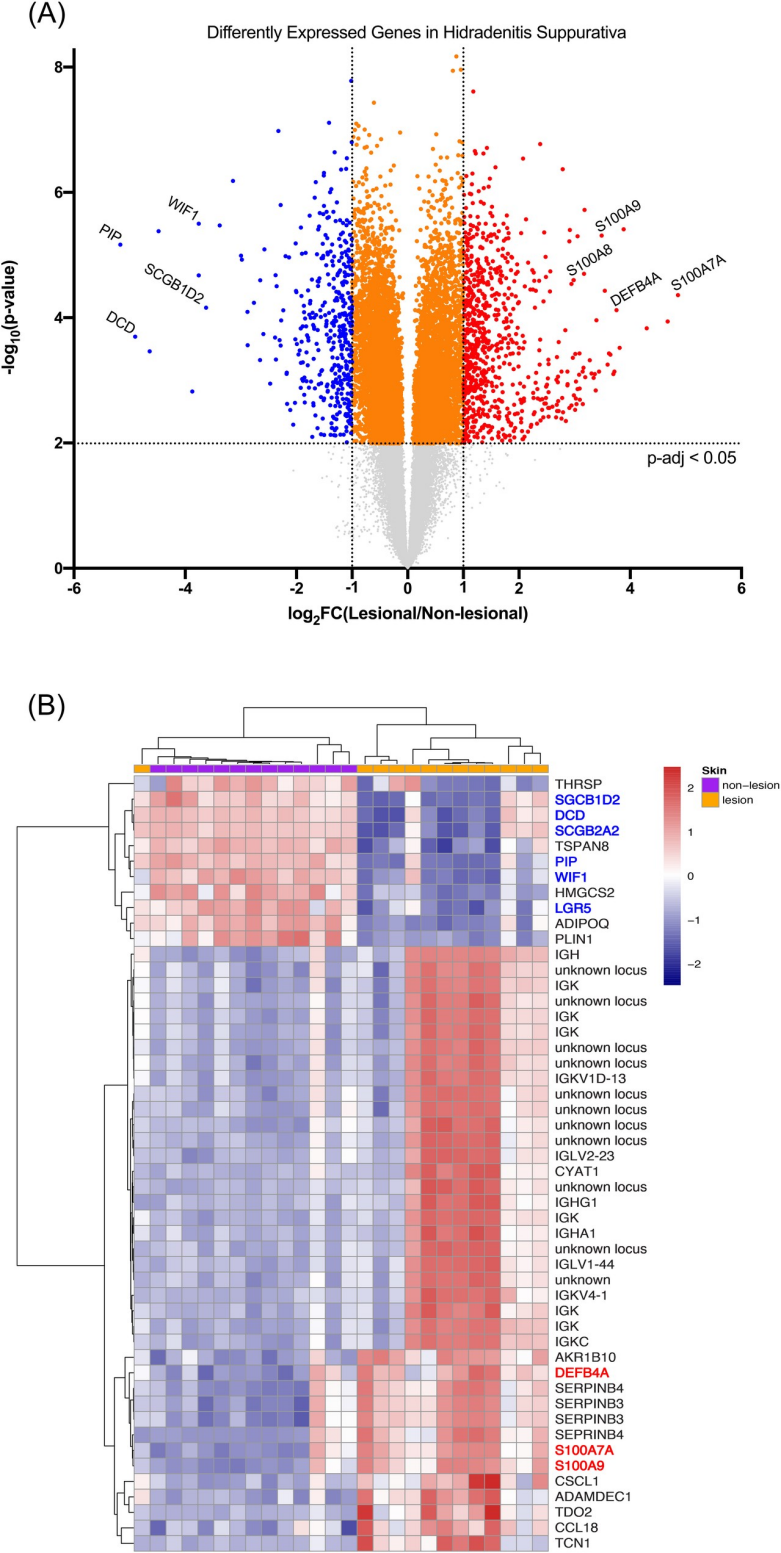
AMPs are increased in lesional HS, but DCD and other sweat-gland associated proteins are decreased. (A) Volcano plot showing increased or decreased genes in HS. Graph shows log FC in gene expression of HS lesional skin over HS non-lesional skin samples plotted against negative log p-value of the difference in gene expression. Genes represented in red are upregulated by >2-fold in HS lesional skin (p-adj < 0.05). Genes represented in blue are downregulated by >2-fold in HS lesional skin (p-adj < 0.05). Genes represented in orange were unchanged (FC < 2, p-adj < 0.05) in HS lesional vs. HS non-lesional skin. Non-adjusted p-values were used for generation of the volcano plot to minimize points with tied y-values but significance level was set using the corresponding non-adjusted p-values. (B) Top 50 most differentially expressed probe sets between the HS lesional skin and the HS non-lesional skin. Highlighting shows DEGs. Genes highlighted in blue are downregulated genes of interest; genes highlighted in red are upregulated genes of interest. While *DCD* is downregulated in HS lesional skin, many other AMPs and interferon-associated molecules are enriched in lesional HS. The top 50 most differentially expressed probes were defined as genes with an adjusted p-value < 0.05 with the largest magnitude FC. Genes were z-score transformed and then the genes and samples were clustered using a correlation distance with complete linkage.

**Fig 2:**
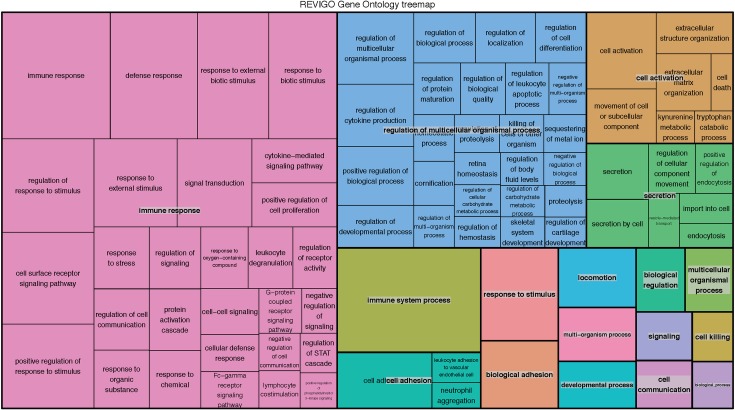
Enriched GO terms. REVIGO treemap representing the most significantly enriched GO terms associated with DEGs [43]. Larger boxes indicate a smaller p-value and greater disease relevance. Colors indicate GO families in which HS DEGs fall.

Analysis of microarray data published in Blok *et al*. comparing HS lesional skin to HS non-lesional skin identified a total of 6,352 DEGs (adjusted p-value < 0.05) (S1 Table). There were 804 unique significant DEGs with a FC > 2 [14]. Of the unique DEGs with a FC > 2, 524 genes were upregulated in the lesional skin relative to the non-lesional skin and an additional 280 genes were downregulated (Fig 1). Gene Ontology Enrichment Analysis (GOrilla) of microarray data revealed a number of Gene Ontology (GO) terms that were enriched [41]. Enriched GO terms included “chemokine production” (GO 0032602), “antimicrobial humoral response mediated by antimicrobial peptide” (GO 0051844), “complement activation” (GO 006956) and “keratinization” (GO 0031424) among others (Fig 2 & Table 2). Top upregulated genes, as previously reported, included AMPs, immunoglobulins, and some keratin types (Table 3) [14, 42]. Top downregulated genes included the AMP *DCD*, genes involved in keratinocytes development and proliferation, and a different subset of keratins (Table 4).

**Table 2:** Enriched GO terms.

GO Term	Description	Relevant genes	Enrichment	FDR p-value
GO 0032602	Chemokine production	S100A8, S100A9	194.54	1.84*10^−3^
GO 0002377	Immunoglobulin production	IGLV1-44, IGKC, IL7R	40.65	4.64*10^−9^
GO 0019731	Antibacterial humoral response	IGHM, IGKV3-20, LTF	40.35	7.83*10^−3^
GO 0050832	Defense response to fungus	S100A12, S100A8, LTF, DCD, S100A9	28.73	1.67*10^−4^
GO 0019730	Antimicrobial humoral response	S100A12, PI3, LYZ, S100A8, S100A7, DCD, S100A9, DEFB4A	27.81	6.81*10^−18^
GO 0051844	Antimicrobial humoral immune response mediated by antimicrobial peptide	S100A12, DEFB4A, S100A7, S100A9	27.45	9.56*10^−9^
GO 0050829	Defense response to Gram-negative bacterium	DEFB4A, LYZ, S100A7	27.15	3.38*10^−4^
GO 0006956	Complement activation	TRBC1, IGHM, C7	18.47	8.76*10^−10^
GO 0031424	Keratinization	KRT19, KRT6B, KRT16, KRT77	6	3.03*10^−3^
GO 0006898	Receptor-mediated endocytosis	ITGA4, SCARA5, IGKC	5.83	2.38*10^−4^

Selected enriched GO terms from among top 100 GO terms with highest enrichment value, where enrichment is defined at (b/n)/(B/N) where N = number of genes total, B = number of genes associated with a given GO term, n = number of genes closer to the top of a ranked list of genes and b = the number of genes in the ranked list that are associated with a given GO term [41]. False discovery rate (FDR) p-values were calculated according to the minimum hypergeometric model corrected for multiple testing using the Benjamini and Hochberg method [35, 41].

**Table 3:** Significant upregulated genes in hidradenitis suppurativa.

Gene	Protein	FC	Function	Reference
S100A7A (S100A15)	Koebnerisin	29.02	Member of S100 family of AMPs and the epidermal differentiation complex (EDC). Induced by *E*. *coli* via TLR4. Markedly increased in psoriatic skin.	[44, 45]
DEFB4A	Beta-defensin 2	13.48	Antimicrobial activity against Gram-negative and Gram-positive bacteria. Has previously been shown to be upregulated in HS.	[32, 46]
S100A9	Calprotecin L1H subunit	11.22	Members of S100 family of AMPs. Stress induced; increased following epidermal injury. Members of the EDC.	[47, 48]
S100A8	Calprotecin L1L subunit	7.69		
PI3	Peptidase inhibitor 3	5.89	AMP against Gram-positive and Gram-negative bacteria and fungi.	[49]
SPRR2B	Small proline rich protein 2B	5.57	Members of the SPRR family of genes in the EDC. Involved in cornified envelope formation.	[50, 51]
SPRR2C	Small proline rich protein 2C	4.75		
KRT16	Keratin 16	5.21	Stress-induced keratin present in wounds.	[52]
S100A7	Psoriasin	4.89	Member of S100 family of AMPs and the EDC. Strongly upregulated in psoriasis.	[53]
S100A12	Calgranulin C	4.18	Member of S100 family of AMPs and the EDC.	[51]
OAS2	Oligoadenylate synthetase 2	3.67	Antiviral protein that degrades viral RNA through formation of 2’-5’ linked oligomers.	[54, 55]
OASL	Oligoadenylate synthetase-like protein	3.40	Antiviral protein that binds viral RNA but lacks classical 2’-5’OAS activity.	
KRT6A	Keratin 6A	2.89	Stress-induced keratin present in wounds.	[52]
LCE3D	Late cornified envelope protein 3D	3.11	Member of the LCE family of genes in the EDC. Expressed late in differentiation in upper granular layers of epidermis. Increased in psoriasis.	[56, 57]

Select upregulated genes in HS lesional skin relative to HS non-lesional skin from among the 200 top upregulated genes. The top 200 upregulated genes are defined as the top 200 unique, significant DEGs (p-adj < 0.05) with the highest positive FC in expression.

**Table 4:** Significant downregulated genes in hidradenitis suppurativa.

Gene	Protein Name	FC	Function	Reference
PIP	Prolactin-inducible protein	0.03	Expressed by sweat glands and also associated with breast cancer.	[58]
DCD	Dermcidin	0.03	AMP with activity against *E*. *coli*, *S*. *aureus*, and *C*. *albicans*. Optimal pH and salt conditions are those found in sweat.	[59]
SCGB2A2	Mammaglobin-A	0.04	Produced in sweat glands. Members of the secretoglobin family are anti-inflammatory. Also likely involved in cell signaling, immune response, and chemotaxis.	[60]
SCGB1D2	Lipophilin-B	0.08		
SCGB2A1	Mammaglobin-B	0.27		
WIF1	Wnt inhibitory factor 1	0.07	Tumor suppressor gene. Inhibits Wnt protein signaling. Involved in sweat gland development.	[61]
LGR5	Leucine rich repeat containing G protein-coupled receptor 5	0.13	Wnt target. Marker of hair follicle stem cells.	[62]
ERBB4	Erb-B2 Receptor Tyrosine Kinase 4	0.20	Epidermal growth factor associated receptor that may play a role in keratinocyte proliferation.	[63]
KRT77	Keratin 77	0.22	Keratin expressed only in eccrine sweat glands.	[64, 65]
KRT19	Keratin 19	0.27	Keratin of simple epithelial cells.	[64]
KRT79	Keratin 79	0.30	Poorly characterized keratin found in skin.	[66]
KRT73	Keratin 73	0.34	Hair follicle-specific keratins.	[64]
KRT74	Keratin 74	0.38		
KRT31	Keratin 31	0.35	Keratin of the hair fiber.	[64]
IL37	Interleukin-37	0.29	Suppressor of innate inflammatory responses. Inhibits dendritic cell activation.	[67]
AQP5	Aquaporin-5	0.31	Water cannel involved in generation of secretions.	[68]
NR1D1	Rev-ErbA-Alpha	0.34	Negative regulator of BMAL1/CLOCK. Involved in regulation of hair follicle cycling.	[69]
PER1	Periodic circadian regulator 1	0.39	Negative regulator of BMAL1/CLOCK.	[70]
FOXA1	Forkhead box A1	0.39	Transcription factor involved in regulation of sweat secretion.	[71]
FOXQ1	Forkhead box Q1	0.43	Transcription factor with role in hair follicle differentiation.	[72]
IL17D	Interleukin 17D	0.44	Pro-inflammatory cytokine overexpressed in psoriasis.	[73]

Select downregulated genes in HS from among the 200 top downregulated genes. The top 200 downregulated genes are defined as the top 200 unique, significant DEGs (p-adj < 0.05) with the most negative FC in expression.

### A number of antibacterial and antiviral proteins are upregulated in HS lesions

The most significantly upregulated gene found in lesional HS skin was *S100A7A* (also known as S100A15, Koebnerisin) (Table 3). *S100A7A* is a member of the S100 family of AMPs, which display antimicrobial activity against gram-negative bacteria, such as *E*. *coli* [44]. Interestingly, *S100A7A* is highly increased in psoriatic skin and shares near-complete homology with *S100A7* (Psoriasin), which has a well-established role in the pathogenesis of psoriasis and is also increased in atopic dermatitis [45, 53]. *S100A7*, which has been shown to be increased in HS lesions in one study, also showed significantly increased expression in our transcriptomic analysis (Table 3) [74]. *S100A8* and *S100A9*, which combine to form Calprotectin, were also among the most increased genes in HS lesional skin [47]. These S100 proteins, which are similarly overexpressed in psoriasis, are also expressed in human wounds, ulcers, and by wound-infiltrating inflammatory cells [48, 75].

**Fig 3:**
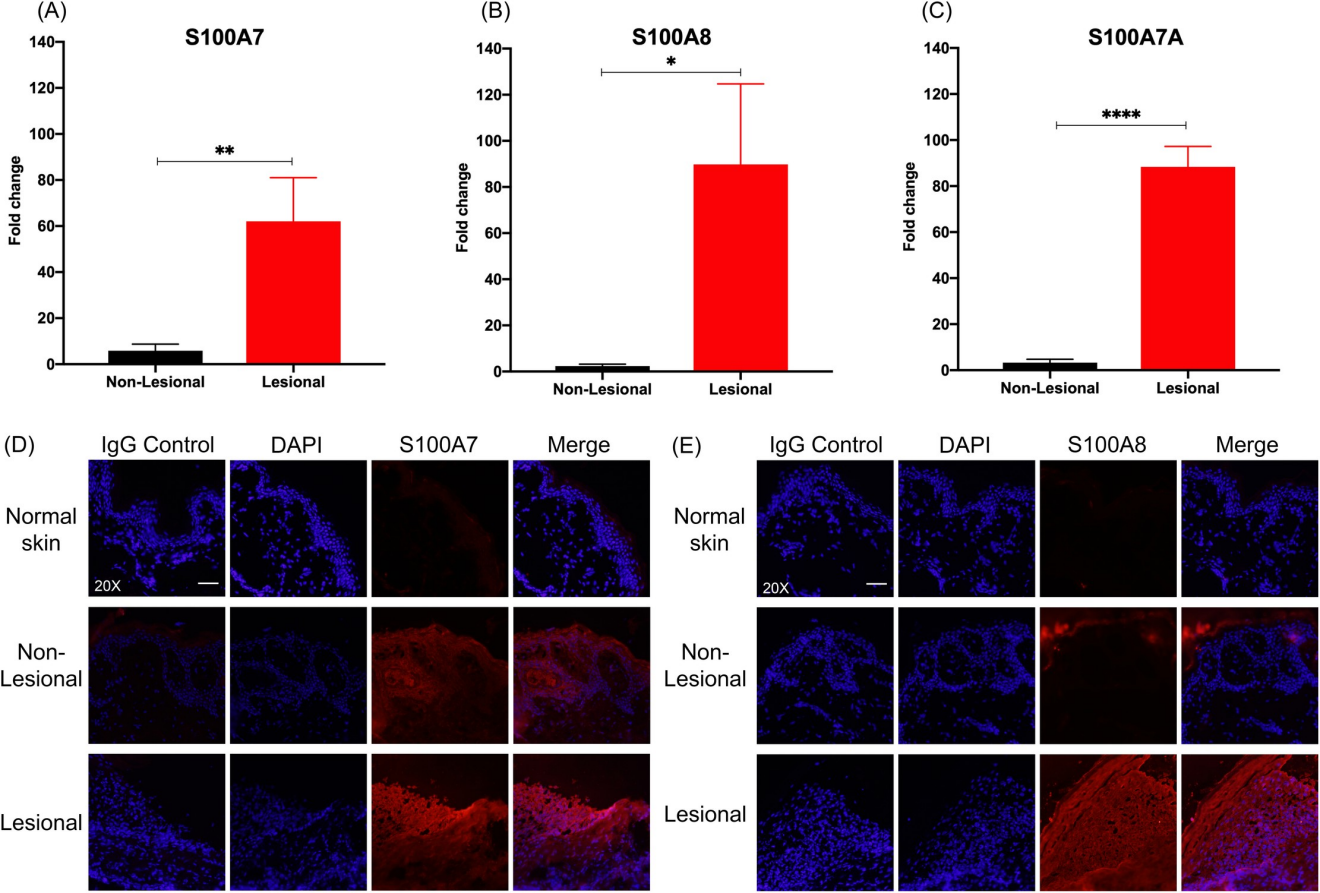
S100 proteins are strongly upregulated in HS lesional skin. qPCR for (A) *S100A7* (log_2_FC = 3.51, **p < 0.01), (B) *S100A8* (log_2_FC = 3.41, *p < 0.05) and (C) *S100A7A* (log_2_FC = 6.47, **** p < 0.0001) in lesional and non-lesional HS skin. FC is expressed as average of skin samples from 3 patients. Measurements were collected in triplicate or duplicate, as allowed by RNA yield from samples. Data is shown as mean expression value compared to non-lesional skin +/- the standard error of the mean. Non-lesional and lesional samples were compared using paired t-test. (D) IF staining for S100A7 at 20X. Scale bar is 50μm. (E) IF staining for S100A8 at 20X. Scale bar is 50μm. Immunofluorescence intensity is highest in HS lesional skin, compared to non-lesional and normal skin.

Expression patterns of S100 proteins were confirmed via qPCR and immunofluorescence on paired samples of HS lesional and non-lesional skin. Consistent with DEG analysis of microarray data, we found that expression of *S100A7*, *S100A8*, and *S100A7A* was significantly increased in HS lesional skin samples (Fig 3).

As previously demonstrated, our analysis of DEGs showed that *DEFB4*, which encodes for human beta-defensin 2 (h-BD2), was strongly upregulated in HS lesional skin (Table 3) [32]. h-BD2 exhibits antimicrobial action against gram-negative bacteria, but not against gram-positive bacteria, such as *S*. *aureus* [46, 76]. Human beta-defensin 3 (hBD-3), which has broad-spectrum antimicrobial activity against gram-negative and gram-positive bacteria, such as *S*. *aureus*, was not significantly increased in HS lesions, which is consistent with prior reported data [77].

Antibacterial proteins are not the only group of AMPs that are increased in HS lesions. Interferon stimulated genes (ISGs), such as *oligoadenylate synthetase 2* (*OAS2*) also had increased expression in HS lesions (Table 3). OAS2, a member of the OAS family of antiviral proteins targets viral RNA primarily through an RNase-L dependent mechanism [54]. OAS family members display potent antiviral activity against both RNA and DNA viruses [24].

### S100 proteins and other members of the epidermal differentiation complex are upregulated in HS

Notably, S100 proteins are members of the epidermal differentiation complex (EDC), a cluster of genes located on human chromosome 1q21 that codes for proteins involved in keratinocyte terminal differentiation and cornified envelope formation [51]. S100A7, which was increased in HS lesions, increases in expression throughout the process of keratinocyte differentiation [78]. S100A8/9 are also upregulated in hyperproliferative epidermis, and levels of S100A8 increase as keratinocytes become more differentiated [51, 75, 78]. Other S100 proteins, such as S100A3, S100A6, and S100A13, which are decreased in differentiated keratinocytes, did not show changes in expression in HS lesions.

Additional members of the EDC had increased expression in HS lesional skin. *Small proline-rich proteins* (*SPRR*) *2B* and *2C* were increased in HS lesions (Table 3). SPRRs participate in cross-bridge formation during development of the cornified envelope of keratinocytes [50]. Late cornified envelope (LCE) proteins are also expressed late in differentiation in the upper granular layers of the epidermis [56]. *LCE3D* is increased in HS lesions; members of the LCE3 group are known to be increased in psoriasis [57].

### Patterns of keratin expression are altered in HS lesional skin

**Fig 4:**
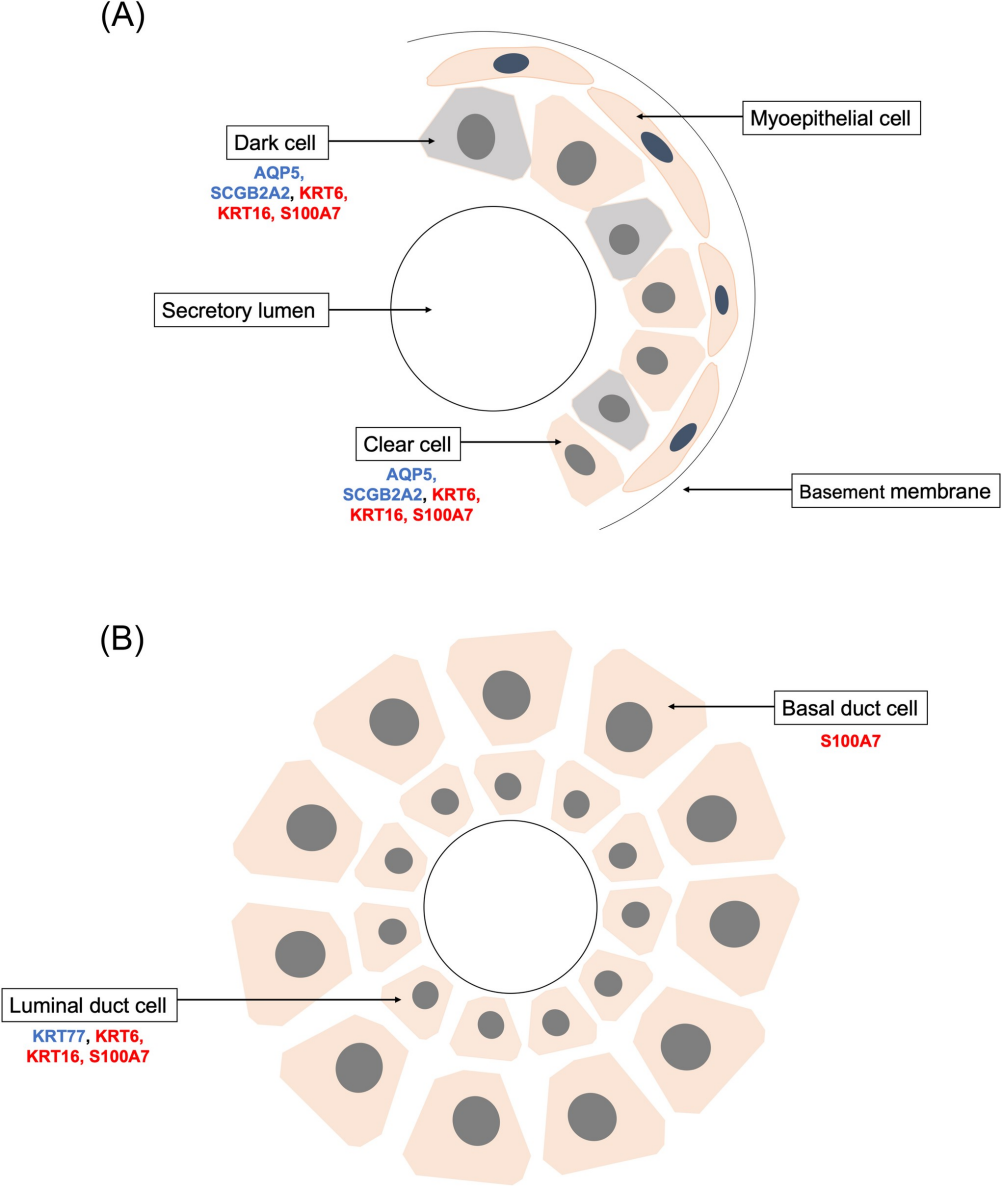
Schematic representation eccrine sweat gland and duct cells. Diagram of gene expression of A) eccrine sweat gland and B) duct. Genes in blue are decreased in HS lesional skin. Genes in red are increased in HS lesional skin.

*Keratins 6A* and *16* are increased in HS lesional skin (Table 3). These keratins, constitutively expressed in the outer root sheath of hair follicles, are increased in multiple inflammatory skin conditions and are makers of a hyperproliferative epidermis [79, 80]. Wounding induces expression of keratins 6 and 16 in the inter-follicular epidermis even before re-epithelialization begins, and keratin 16 may play a role in reorganization of other keratin filaments during healing [52, 81]. However, mice overexpressing keratin 16 have delayed wound healing [82]. Notably, keratins 6A and 16 are also expressed in secretory and luminal cells of eccrine sweat glands (Fig 4) [83]. Increased expression of these keratins in HS lesions is not due to the number of eccrine sweat glands in the adnexal and inguinal regions, where HS is often found; *keratin 77*, which is exclusively expressed in eccrine sweat glands, is here shown to be decreased in HS lesions (Table 4) [65]. Other keratins are also decreased in HS lesions, including *keratin 73* and *keratin 74*, which are specific to the hair follicle [64].

### DCD, a sweat-gland associated AMP, is decreased in HS lesions

In drastic contrast to the large number of AMPs that were strongly upregulated in HS skin, we identified that *DCD* is one of the most significantly downregulated genes in HS lesions (Table 4). DCD is an AMP secreted by sweat glands to provide antimicrobial function against bacteria and a broad spectrum of microbes including fungi and viruses [84]. While most AMPs carry a strong positive charge, and prefer to bind to bacterial membranes, DCD has an overall negative charge, so it relies on positively-charged zinc ions, which are abundant in sweat, to assist with its specific interaction with bacterial lipids [59]. In human healthy skin, DCD is known to be expressed in the dark mucous cells of the secretory coil of eccrine sweat glands and is found in the Golgi complex and the secretory granules typical for a secreted protein [59].

DCD is a relatively newly discovered AMP, so its role in skin protection and/or disease is not yet fully understood. DCD is proteolytically processed into multiple peptides, including DCD-1L, which exhibits antimicrobial activity [85]. Y-P30, another peptide product of DCD, is considered a “survival-promoting peptide” for neurons [84]. The antimicrobial activity of DCD is broad and includes both gram-positive and gram-negative bacteria, fungi, and even viruses [84]. DCD is constitutively secreted, which suggests that it may play a role in maintaining a favorable skin microbiome under homeostatic conditions. This is in contrast to other AMPs, such as LL-37, which are induced by bacteria and wounding [26].

**Fig 5:**
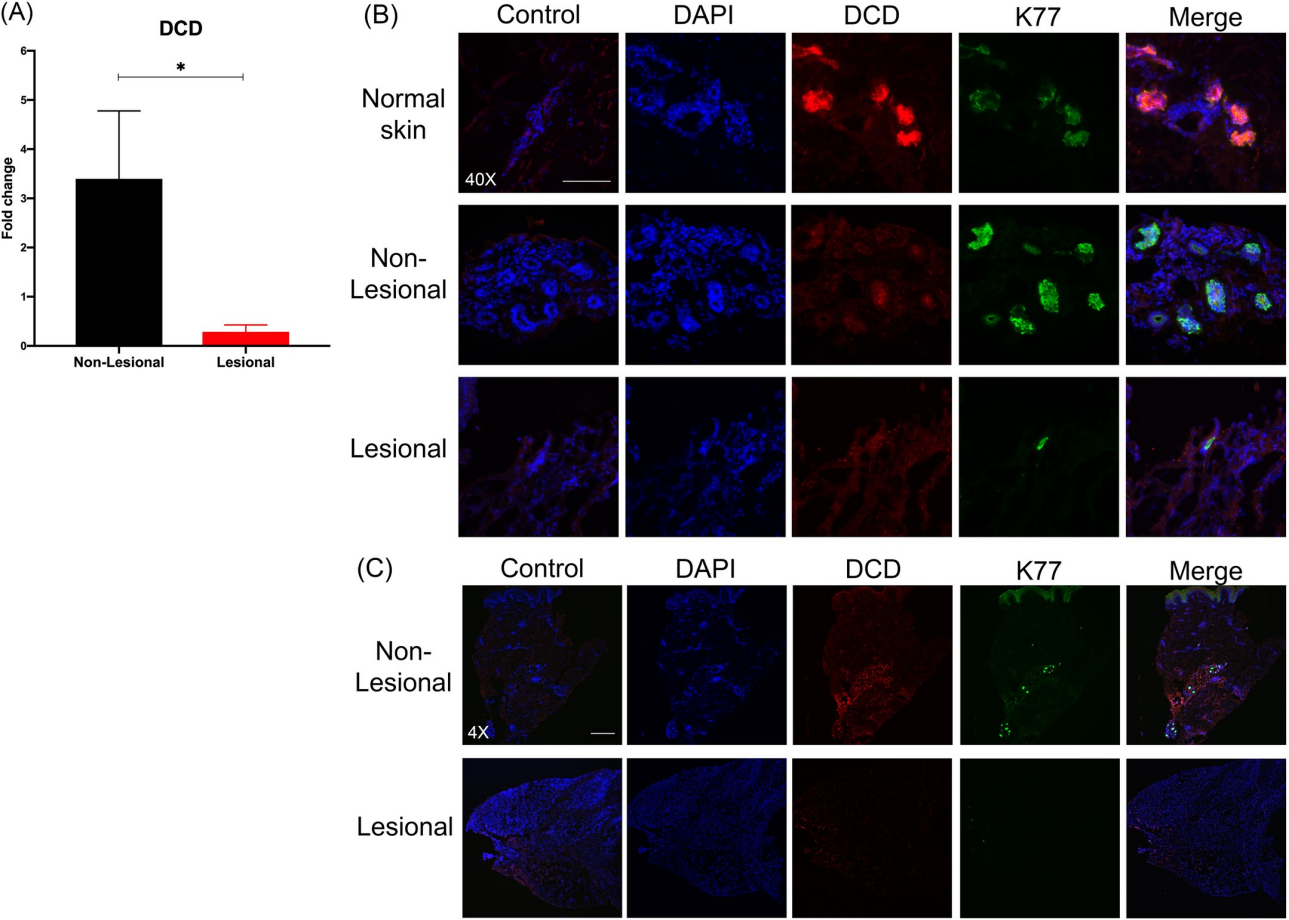
DCD is strikingly decreased in HS lesions. (A) qPCR for *DCD* (log_2_FC = -3.57, *p < 0.05). FC is expressed as average of skin samples from 3 patients. Measurements were collected in triplicate or duplicate, as allowed by RNA yield from samples. Data is shown as mean FC over non-lesional skin +/- the standard error of the mean. Non-lesional and lesional samples were compared using a paired t-test. (B) Immunofluorescence for DCD (red) at 40X in normal skin, HS non-lesional skin, and HS lesional skin. Co-staining was performed with K77 (green), which is a marker of eccrine sweat glands. Scale bar is 100μm. (C) Immunofluorescence for DCD (red) and K77 (green) at 4X showing decreased number of eccrine sweat glands in HS lesional skin. Scale bar is 500μm.

Given the strong downregulation of DCD in HS lesions, in contrast to the increase of most other AMPs, we sought to further elucidate the role of DCD in HS. Analysis via qPCR confirmed the significant decrease of nearly 12-fold in *DCD* in HS lesional samples (Fig 5A). Furthermore, we used immunofluorescence to examine expression of DCD in matched HS non-lesional and lesional skin from a single patient. Fluorescence intensity for DCD was stronger in non-lesional skin, compared to lesional skin (Fig 5B). In addition to the decreased fluorescence intensity of DCD exhibited by HS lesional skin, we also observed that fewer eccrine sweat glands (as marked by K77) were found in HS lesional skin than normal skin or HS non-lesional skin (Fig 5C).

Correlation analysis was run to determine genes positively and negatively correlated with the *DCD* probe set (Table 5) in the Blok *et al*. dataset (S2 Table) [14]. Top correlated genes with *DCD* included members of the *secretoglobin* family (r = 0.97, 0.99; adj p-value = 3.25x10^-18^, 2.34x10^-11^), *WIF1* (r = 0.87, adj p-value = 9.90x10^-6^), and *AQP5* (r = 0.79, adj p-value = 1.84x10^-4^), which were some of the most downregulated genes overall in HS. On the other hand, *DCD* was negatively regulated with multiple molecules from the interferon and antiviral protein pathway, such as *STAT1* (r = -0.88, adj p-value = 7.78x10^-6^), *IRF1* (r = -0.84, adj p-value = 6.93x10^-5^), *TLR8* (r = -0.79, adj p-value = 1.15x10^-4^), *and IFNAR2* (r = -0.74, adj p-value = 8.41x10^-4^). IFNAR2 signals through a STAT-dependent mechanism to turn on production of ISGs, which have antiviral activity [86]. Findings from the correlation analysis were mirrored by expression analysis in the microarray data set, where both the *interferon receptors 1* and *2* (*IFNAR1* and *2*) and *OAS2*, a downstream ISG, were upregulated in lesional HS skin relative to non-lesional skin.

**Table 5:** Selected genes most positively- or negatively-correlated with DCD.

Gene	Protein Name	Correlation with DCD	Function	Reference
DCD	Dermcidin	1	AMP with activity against *E*. *coli*, *S*. *aureus*, and *C*. *albicans*. Optimal pH and salt conditions are those found in sweat.	[59]
SCGB2A2	Secretoglobin 2A2	0.99	Produced in sweat glands. Members of the secretoglobin family are anti-inflammatory. Also likely involved in cell signaling, immune response, and chemotaxis.	[60]
SCGB1D2	Secretoglobin 1D2	0.97		
WIF1	Wnt inhibitory factor 1	0.87	Tumor suppressor gene. Inhibits Wnt protein signaling. Involved in sweat gland development.	[61]
AQP5	Aquaporin 5	0.79	Water cannel involved in generation of secretions.	[68]
STAT1	Signal transducer and activator of transcription 1	-0.88	Transcription factor involved in upregulation of genes following type I, II, or III interferon signaling.	[87]
SAR1B	GTP- binding protein SAR1b	-0.84	Sar1-ADP ribosylation factor family of small GTPases, which govern the intracellular trafficking of protein in coat proteins (COP)-coated vesicles. Plays a role in clathrin-mediated endocytosis signaling.	[88]
IRF1	Interferon regulatory factor 1	-0.83	Regulates expression of target genes by binding to an interferon stimulated response element in their promoters. May contribute to multiple autoimmune diseases.	[89]
TLR8	Toll-like receptor 8	-0.81	Recognizes single stranded viral RNA. Activation of TLR8 can initiate development of psoriatic lesions.	[90]
RAB31	Ras-related protein Rab-31	-0.79	Member of the RAS oncogene family. Small GTP-binding protein. Has a role in targeting of vesicles and granules.	[91]
IFNAR2	Interferon receptor	-0.74	Receptor for type I IFN. Activation of the receptor stimulates Janus protein kinase (JAK), which in turn phosphorylate STAT1/2. May even have intrinsic antiviral activity.	[92]

Sweat gland proteins were among the genes most positively correlated with DCD in HS skin samples (all statistically significant with an adjusted p-value < 0.05). Signaling molecules of the interferon and antiviral protein pathways were negatively correlated with DCD.

### Proteins associated with sweat gland function are decreased in HS lesions

A number of genes related to sweat gland function had differential expression in HS lesions. Expression of genes coding for S100 proteins, which are present in multiple sweat glands cell types, was increased (Table 3) [93]. In contrast, expression of a number of other sweat-gland associated proteins was decreased. *Secretoglobins B2A2*, *B1D2*, and *B2A1* were among the genes with the largest decrease in expression in HS lesions (Table 4 and Fig 4). Secretoglobins, for example, may play a role in sweat secretion [60]. *Wnt inhibitory factor 1* (*WIF1*), an inhibitor of Wnt signaling, was decreased in HS lesional skin. Dynamic and tightly regulated expression of Wnt, Shh, and Eda has been previously linked to sweat gland development and function [94]. *Aquaporin 5* (*AQP5*), which contributes to sweating by increasing permeability of sweat glands, was also decreased in HS lesions [68, 95]. Finally, we found that *forkhead box A1* (*FOXA1*), a transcription factor that is required for sweat secretion in mice, is decreased in HS lesions [71]. In sum, we found that multiple genes involved in development, regulation, and homeostasis of sweat glands show decreased expression in HS lesions.

Interestingly, genes involved in hair follicle differentiation and proliferation were also decreased in HS lesions (Table 4). For example, the *leucine-rich repeat containing G protein-coupled receptor 5* (*LGR5*) was decreased in lesional skin. LGR5 is a Wnt target gene that is a marker of proliferating stem cells in the hair follicle [62]. Additionally, *forkhead box Q1* (*FOXQ1*), a regulatory target with a role in hair follicle differentiation, was also decreased [72]. Expression of hair follicle-associated genes, in addition to sweat gland-associated genes, is altered in HS.

### HS lesions share a transcriptomic signature with wounds

Notably, the most severe HS lesions (Hurley stage III) are characterized by chronic non-healing sinuses, which form a wound-like environment [96]. Previous understanding of wound closure was that new skin cells originate from hair follicles and from intact skin at the edge of the wound [97]. More recent studies demonstrated that cells also arise from beneath the wound, and suggested that human eccrine sweat glands also store an important reservoir of adult stem cells that can quickly be recruited to aid wound healing [98]. Based on our findings that genes related to sweat gland development and re-epithelialization were downregulated in HS lesional skin, we compared the transcriptomes of HS skin with wounded skin.

**Fig 6:**
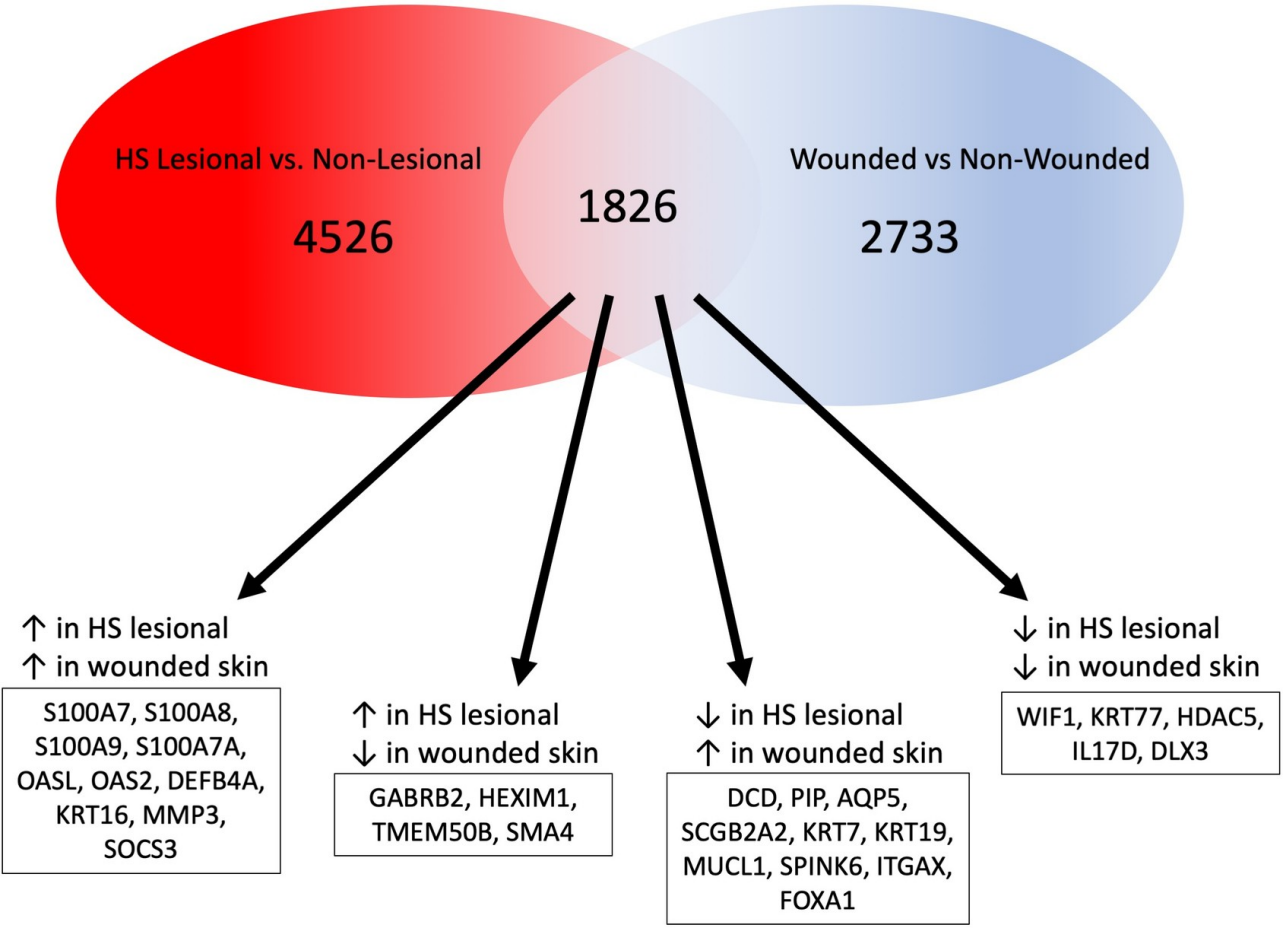
Shared and dissimilar pathways in HS and healing skin wounds. Venn diagram illustrating shared and dissimilar gene expression in HS lesions and wounded skin. AMP expression is increased in both HS lesional and wounded skin. DCD and other sweat-gland associated proteins show opposite expression; they are decreased in HS lesions but are increased in wounded skin.

Re-analysis of wounded skin vs. non-wounded skin from Iglesias-Bartolome *et al*. revealed 4,599 DEGs (adjusted p-value < 0.05) (S3 Table) [31]. 1,826 of these genes were also differentially expressed in HS lesional skin compared to non-lesional skin (Fig 6). A number of the shared DEGs were increased in both HS lesional skin relative to non-lesional skin and in wounded skin relative to non-wounded skin. These include S100 family members (*S100A7*, *S100A8*, *S100A9*, and *S100A7A*), *DEFB4A*, the ISGs *OASL* and *OAS2*, and *KRT16*. The shared increased expression of antibacterial and antiviral proteins by HS lesions and wounded skin suggests that they are carrying out similar antimicrobial programs. Stress-induced *keratins 6* and *16* were upregulated in both HS lesions and wounds [52]. Other shared DEGs were decreased in both HS lesional skin and wounded skin, including *WIF1*. Finally, a small number of genes showed opposite expression in HS lesions and wounded skin. Among these genes were *DCD*, *AQP5*, and *SCGB2A2*. Whereas these genes were upregulated in wounded skin, they were downregulated in HS skin, suggesting that they play a role in the pathology of HS, but are beneficial for wound healing.

## Discussion

The striking increase in expression of many AMPs in HS lesions brings forth two hypotheses for pathogenesis of HS. Firstly, increased AMP expression could be the result of a general overactivation of the innate immune system in response to bacteria or other stimuli. Secondly, the increased AMP expression could be a reaction to an altered cutaneous microbiome of HS lesions. Whether changes in AMP expression are inherent to the disease or secondary to the altered microbiome of HS, dysregulation of AMPs may contribute to the initiating pathogenesis of HS or contribute to disease progression and aggravation. HS lesions are characterized by upregulation of defensins, a group of AMPs that target gram-negative bacteria, but not gram-positive bacteria, and this could provide a rationale for the increased prevalence of gram-positive microbes, particularly *S*. *aureus*, in HS lesions [99]. Although more research is needed to address these questions, our work shows that there is significant dysregulation of AMP expression in HS lesions.

A second theme that emerged from re-analysis of microarray data from *Blok et al*. is that many of the genes that were upregulated in HS are members of the EDC [14]. Interestingly, genes of the EDC were the focus of a recent study comparing the genes expression profiles of mice raised in the presence of commensal microbiota (specific pathogen free, SPF) with mice raised in a germ-free environment [100]. Meisel and colleagues found that S100A7 and many LCE proteins were increased in SPF mice. Based on the increased expression of these EDC genes, which are makers of terminal differentiation, it was hypothesized that SPF mice have decreased regenerative capacity compared to GF mice. Specifically, our findings suggest that there may be a greater number of terminally differentiated keratinocytes in HS lesions. Therefore, HS lesional skin may have less regenerative capacity than normal skin. Therefore, although significant research will be required for definitive conclusions to be drawn about the role of the EDC in HS, our analysis indicates that epidermal differentiation and regeneration pathways could be involved in the pathology of HS.

There is strong evidence emerging that human sweat glands contribute significantly to epidermal homeostasis and wound repair. It is well known that stem cells in the hair follicle bulge contribute to re-epithelialization [97]. More recently, it has been shown that eccrine sweat gland cells are able to reconstitute a stratified interfollicular epidermis with all features characteristic of a normal stratified squamous epithelium both *in vitro* and in rat models [101]. These findings have been replicated in humans using immunohistochemical staining of healing skin wounds [98]. In fact, there are distinct, multi-potent, stem progenitor cell populations residing within sweat glands [102]. In elderly skin, re-epithelialization by sweat gland cells is impaired and may account for poor wound healing in this population [103]. Severe HS lesions resemble chronic, non-healing wounds and are characterized by sinus tracts, scarring, abscesses, and bacterial superinfection [96]. Given that sweat glands and sweat gland function are integrally important for wound healing, it is possible that impaired sweat gland function and decreased sweat gland number contribute to the pathological non-healing wound-like environment of HS. Our analysis determined that multiple genes associated with sweat-gland function, such as *WIF1*, *AQP5*, *FOXA1*, and *DCD* were decreased in HS lesions. Wnt signaling is required for development of sweat glands, suggesting that sweat gland development may be impaired in HS [61]. WIF1 is also decreased in psoriatic skin [73]. AQP5 is a transmembrane protein that increases water permeability of cells, and therefore contributes to sweat formation [68, 95]. Knockout of AQP5 function impairs sweat secretion in mouse models, raising the possibility that the decreased expression of *AQP5* in the eccrine glands of HS lesional skin impairs sweat generation [95]. Impaired sweat gland function via downregulation of these key sweat gland-associated genes could contribute to HS.

Impaired sweat secretion could be one reason for the reduced levels of DCD in HS lesions. However, a second possibility for decreased DCD level in HS lesional skin is a decreased overall number of eccrine sweat glands in HS lesions. We showed via immunofluorescence that fewer eccrine sweat glands are present in HS lesional skin than normal skin or HS non-lesional skin. Moreover, staining intensity of DCD in HS lesions within the HS lesional skin samples was also reduced compared to those found in donor-matched healthy non-lesional skin or healthy normal skin. This raises the possibility that the decreased gene expression levels of *DCD* seen in transcriptomic and qPCR data may be due to a combination of decreased DCD production by eccrine glands and a fewer number of total eccrine glands in HS lesional skin. Loss of normal skin architecture can be seen in HS, which may contribute to the decreased number of eccrine sweat glands observed in HS lesional skin samples [30].

Furthermore, we found that expression of many sweat gland-associated genes was different in HS and acute healing wounds. Wound samples in the RNA-seq data set from Iglesias-Bartolome *et al*. were acquired from the axillary region of subjects, therefore the density of eccrine glands in the wound samples is representative of wounded healthy skin from areas where HS often occurs [31]. It is important to note that the wound data that was used for overlap analysis with HS samples is from wounds that ultimately went on to heal, indicating that genes that were increased in this data set could be important for healing. Genes such as *AQP5*, *FOXA1*, and *DCD* were increased in acute wounds, but were decreased in HS. The finding that sweat gland-associated genes were decreased in HS lesional skin, which resembles a chronic non-healing wound, points to these genes as potential pathogenic explanations for the HS phenotype.

## Conclusion

Hidradenitis suppurativa is a chronic and frequently debilitating cutaneous disorder that significantly impacts the quality of life of patients. Compared to other cutaneous disorders, such as psoriasis and atopic dermatitis, it is relatively poorly characterized. HS is significantly different from these classic inflammatory skin diseases through the clinical presentation of non-healing skin lesions and the formation of ducts and cysts that become highly inflamed. Our transcriptomic analysis of HS lesions suggests a significant role for innate antimicrobial immunity and altered sweat gland function in HS disease pathology and furthermore revealed a previously unknown set of DEG that overlap with healing wounds.

Recent studies have begun to illuminate the role of sweat gland cells, specifically sweat gland progenitors, in wound healing and re-epithelialization. Sweat glands contain multipotent progenitor cells that can migrate to epidermal layers of the skin and contribute to repair; in addition, eccrine ductal cells participate in re-epithelialization [98, 102]. Sweat glands may also contribute to cutaneous immunity beyond their role in wound repair through production of inflammatory cytokines and DCD, a sweat-gland associated AMP [59, 104]. Therefore, it is possible that sweat glands produce multiple host factors, including antimicrobial DCD that promote epithelial regeneration and that this pathway is dysregulated in HS prohibiting healing of severe HS lesions.

We also uncovered substantial transcriptional overlap between HS lesions and wounded skin, suggesting that HS may represent a wound-like environment. Our analysis could pave the way for development of new therapies for HS. For example, supplementation and activation of natural AMPs, such as DCD, may be promising therapeutic options for the treatment of HS.

## Supporting information

S1 Table HS skin transcriptome analysis of GSE72702. (XLSX)

S2 Table DCD correlation probe. (XLSX)

S3 Table Wound healing over time analysis of GSE97615. (XLSX)
